# Lessons Learned from Providing Integrated Care Within an Interprofessional Learning and Innovation Network in the Community

**DOI:** 10.5334/ijic.9122

**Published:** 2026-06-25

**Authors:** Petra Boersma, Lilian Linders, Henk L. G. R. Nies, Robbert J. J. Gobbens

**Affiliations:** 1 Faculty of Health, Sports and Social Work, Inholland University of Applied sciences, Pina Bauschplein 4, 1095 PN, Amsterdam, the Netherlands; 2 Ben Sajet Centrum, Amsterdam, the Netherlands; 3 Faculty of Health, Sports and Social Work, Inholland University of Applied sciences, Bijdorplaan 15, 2015 CE Haarlem, the Netherlands; 4 Faculty of Social Sciences, Organisation Sciences, VU University, De Boelelaan 1105, 1081 HV Amsterdam, the Netherlands; 5 Zonnehuisgroep Amstelland, Amstelveen, the Netherlands; 6 Tranzo, Tilburg University, Tilburg, the Netherlands; 7 Department Medicine and Population Health, Faculty of Medicine and Health Sciences, University of Antwerp, Antwerp, Belgium

**Keywords:** interprofessional collaboration, interprofessional learning, integrated care, nursing, social work, community care

## Abstract

**Introduction:**

The Interprofessional Learning and Innovation Network (IP-LIN), comprising nursing and social care professionals, students, and teachers, was established to enhance integrated care competences, specifically attention to health and wellbeing for older adults living in the community.

**Description:**

This case study with ten interprofessional case discussions and two focus groups was completed with nursing and social work professionals, students and teachers. Thematic analysis regarding five interprofessional competences of the Zuyd Interprofessional Building Blocks model was conducted. Key recurring themes included responsibility, available time, mutual understanding of professional language, reflection on attitudes, and optimising client autonomy. Identified barriers were the absence of a joint care plan, incomplete application of the methodical circle, and insufficient consultation among professionals involved with the client.

**Discussion:**

Participants recognised that interprofessional learning fosters improved collaboration between nursing and social work domains; however, the absence of a joint care plan remains a significant obstacle to fully integrated care.

**Conclusion:**

The IP-LIN facilitated opportunities for professionals, students, and teachers to understand each other’s roles, responsibilities, and perspectives, thereby increasing confidence in interprofessional and team competencies. Successful IP-LIN implementation requires sustained commitment from both care and social work sectors to overcome existing barriers and promote integrated care.

## Introduction

The world population is expected to age significantly, as is the European population. The United Nations Department of Economic and Social Affairs predict that in 2050, 34% of the European population may be over 60 years old [1]. Ageing is related to frailty, disability, and multimorbidity [2] with a higher risk of adverse outcomes such as falls. This often results in complex care needs [3,4]. In public health, multimorbidity is recognised as a major challenge [5], not only due to multiple chronic diseases, but also because older adults suffer from mental health problems and social vulnerabilities such as loneliness and lack of participation [6]. This places pressure on the integration of, amongst others, primary care and social care professionals (from here onwards referred to collectively as community care). Community care professionals have the challenge of paying attention to the physical as well as the social and mental aspects of older adults living in the community. This integrated care^1^ [7] for older adults living in the community, involves multiple domains of human functioning. Interprofessional collaboration (IPC) is defined as ‘an active and ongoing partnership between people from diverse backgrounds with professional cultures and possibly representing different organisations or sectors who work together to solve problems or provide services’ [8], and contributes to integrated care. It has been known for some time that IPC makes a positive contribution to people living in the community, for instance for people with chronic and complex multimorbidity [9]. IPC has demonstrably improved patient safety, patient satisfaction [10], health care quality and health outcomes [11]. It has also improved job satisfaction for interprofessional caregivers [10]. Although the WHO interprofessional collaboration recommends [12], and researchers and professionals believe that IPC has this important added value, its implementation and evidence are still in their infancy [13]. For the Netherlands, Kromhout et al. reported that the mutual relationship and coordination between the different forms of care and support (formal, informal) required to provide integrated care have not yet sufficiently gotten off the ground [14]. There is still little access to integrated care and little evidence of innovative, integrated care [14]. To change this situation education and practice innovation is needed. To strengthen integrated community care and to improve interprofessional learning and collaboration between students, professionals, and teachers of the nursing and the social care domain, an Interprofessional Learning and Innovation Network (IP-LIN) was set up. Albers et al. [15] described a Learning and Innovation Network (LIN) as:

*“a group of care professionals, students and education representatives who come together in clinical practice and are all part of a learning and innovation community in nursing. They are active members who shape their own learning process and support other learners. They constantly reflect and learn from and with each other by a combination of individual and team learning activities. They work together on practice-based projects in which they combine best practices, research evidence, and client perspectives (Evidence-Based Practice) in order to innovate and improve the quality of care and in which an integration of education, research and practice takes place”*.

The LIN is closely related to Care Innovation Units (CIUs), where qualified nurses, students and teachers collaborate to integrate care, education, innovation and research [16]. The LIN also has affinities with the concepts of Dedicated Education Units (DEUs) and the Nursing Development Units (NDUs) [17,18,19].

## Ethical approval

The ethical commission of Zonnehuisgroep Amstelland approved this study. Integrity of the respondents was not encroached upon as a consequence of participating in this study, which is the main criterion in medical–ethical procedures in the Netherlands [20]. Nevertheless, all participants, included older adults living in the community were informed orally and in writing prior to the study by professionals or students and provided written informed consent. The older adults could participate in this project without any specific preparatory training or support.

## Description of the Interprofessional Learning and Innovation Network

The IP-LIN brings together different practices, e.g. the nursing and social work professionals on the one hand and the ‘school’ on the other. The IP-LIN forms a ‘hybrid workplace learning’ in which participants may experience sociocultural differences as boundaries [21]. While these boundaries can disrupt interactions, they also offer valuable learning opportunities through boundary crossing [22]. As mentioned before and explained below, innovation and research are integral to the IP-LIN.

The IP-LIN includes intermediate and higher vocational nursing and social work students, professionals, and teachers. Students join in four consecutive periods, completing twenty-week internships. Weekly four-hour meetings bring together students, professionals, and teachers to engage in three activities fostering social learning, innovation, reflection, and co-production [16]: a) working together on small *practice-based projects*, b) *reflection* to stimulate competence development, and c) *interprofessional case discussions*. The executed *practice-based projects* were: supporting informal caregivers to prevent overburdening, loneliness, hygiene issues, and use of smart technology to enhance client self-reliance. These topics were jointly determined at the start of each period. Once chosen they designed a small project plan with different research activities, e.g. literature research, interviewing clients or their informal carers, interviewing professionals, trying out and evaluating new methods or instruments. At the end of the period they presented the results of their practice based project to the whole team and organisational staff. Teachers facilitated *reflection* through peer-group coaching, and supporting professional competence development as nurses or social workers. The third activity, the *interprofessional case discussions* were held with the aim to stimulate and experience real life interprofessional learning and collaboration between nursing and social work students, professionals and teachers. In these conversations, the focus was on discussing a current complex client situation in the community, which nurses and/or social workers had questions. The next section describes the case discussions in detail. Together, these LIN-activities strengthen interprofessional teamwork, enabling integrated care aligned with current standards and improving care quality [17].

### Interprofessional case discussions

Between June 2019 and July 2021, ten interprofessional case discussions were conducted involving nursing and social work professionals, students, teachers, and the first author/researcher (PB). Two weeks prior to each case discussion, the professionals and students jointly selected a client from their caseload, based on a certain degree of case complexity or specific interprofessional learning questions. Clients were invited by professionals or students to participate. A student or professional supported the client with online participation and by explaining questions during the case discussion. The role of clients was to clarify their needs, share their experiences with the care they received, and critically assess the integrated care proposals that were made during the discussion. The researcher (PB) acted as a participatory observer; she participated and observed as part of the research. The case discussion method [23] followed six steps: 1) case insertion; 2) clarify the case; 3) participants determine their individual approach; 4) group discussion; 5) conclusions; and 6) evaluation. Due to the COVID-19 pandemic, seven case discussions were held online via Microsoft Teams. In box 1 an anonymous vignette of a case discussion is described. Although this study was conducted about six years ago, the IP-LIN is still active, with ongoing interprofessional case discussions. Continued collaboration between care and social work professionals and students remains essential to improve clients’ quality of life.

**Box 1 Anonymised vignette of a case discussion**

In this case discussion, the client, two teachers, three district nurses, two social workers, one social work student, four nursing students, and the researcher participated. After the introduction of the clients’ situation by the district nurse (he suffers from Parkinson’s disease, which for the last year has deteriorated him physically, neurologically, and cognitively, making him more dependent on his two children), all start asking questions to the client to clarify the case. The client explained that his deepest wish was to take medicine for Parkinson’s disease, but he knows this doesn’t exist. Furthermore, he said he was very grateful for the support. However, there is room for improvement in communication between the different care groups throughout the day as not everyone is informed about the latest developments. He feels a loss of independence; a few years ago, he was still driving a car and running errands. He misses this. He hates his new bed (against incontinence), because the nurses bring him to his bed early in the evening, so he is no longer able to watch television and misses being informed from the television as well. Previously, he enjoyed certain learning courses via computer, for example, in law and criminal law. However, at the time, he did not have a computer. The social worker suggests arranging an iPad so he can watch television in bed and can also help with participating in online lectures. The nurse also suggested moving his bed so that he could watch television in the evenings. The student social work remarked that the client missed visiting the grocery store and suggested that the client go to the supermarket with a volunteer every two weeks, so that he could choose his own groceries. ‘Instead of using a walker, you can also use a wheelchair’. The Teacher summarises the opportunities and asks the client what he thinks about them. He reacts positively and is interested. They agree that the nurse and social worker will work on these options with the client and also on their request for better cooperation between the different visiting caring groups. The discussion ended with a short evaluation: all professionals and students were very positive about this session. ‘You get other ideas.’ ‘It is very instructive for us as a team.’ ‘There were asked very good questions and we got a better understanding of how the client experiences his day and what is important to him.’ The client reacted ‘Look, I am obviously going through a very uncertain period. I do not know at all how it will turn out, but I want to thank you all.’ The social worker closed: Different options must be examined calmly and worked out.

## Methods

### Study aim and design

The aim of this study was to gain insight into the interprofessional learning and collaboration processes in an IP-LIN in the community, in order to improve integrated care to independently-living vulnerable older adults. A case study [24] was performed in two Dutch long-term care organisations who provide nursing care and one social work organisation who provides social care to older adults living in the community in the Amsterdam region.

### Data collection

Qualitative data was collected with (audio) recordings of three live case discussions and seven online discussions via Microsoft Teams. At the end of the project, two online focus groups were held. Purposive sampling was used to select the participants so that a mix of (nursing and social work) professionals, teachers, and students participated. The topics of the focus groups were derived from The Consolidated Framework for Implementation Research (CFIR) [25] and translated in fourteen statements (see supplementary file), which were drawn up by the project leader (RG), the researcher (PB), a teacher (CdB), and a client representative. All participants of the case discussions and the focus groups had participated for a minimum of half a year in an IP-LIN. All recordings were transcribed.

### Data extraction and analysis

The verbatim transcripts of case discussions and focus groups were analysed thematically and deductively [26]. Unlike the data collection in which the CFIR theory [25] was used to formulate the fourteen statements, the researchers (RG; PB) analysed following the competences for interprofessional cooperation from the Zuyd Interprofessional Building Blocks model [27], because this model better matched the aim of the study, which also focused on interprofessional learning and working. The data was coded based on its five blocks with the different competences required for interprofessional learning and working: 1) Collaborate and understand (11 items); 2) Work out interprofessional care plans (8 items); 3) Deal with problems (8 items); 4) Make appropriate referrals (5 items); and 5) Evaluate (6 items). The detailed description of these blocks can be found in the supplementary file. To maintain rigor, analyses of the case discussions were independently performed by the researcher (PB) and teacher (CdB), and analyses of the focus groups were independently performed by the researcher (PB) and project leader (RG) and subsequently discussed [28]. The results were summarised in matrices, categorising themes concerning the interprofessional learning and collaboration between nursing and social work students, professionals and teachers. Afterwards, all identified themes were discussed with three members of the project group (project leader (RG), researcher (PB), client representative). To support the qualitative data analysis, MaxQdata 2020 (www.maxqda.com) was used. The themes are presented in italics in the text and are illustrated with verbatim quotes from case discussions (C) and focus groups (FG) with numbers and role of respondents.

## Results

### Characteristics of the participants of IP-LIN

Twenty-three participants from nursing and social work – professionals, teachers, and students – took part in the IP-LIN. One secondary vocational student dropped out due to ending her internship, and one nurse and one social carer withdrew after changing jobs. Table 1 details participant characteristics.

**Table 1:** Characteristics of the participants of the IP-LIN.


CHARACTERISTICS		PARTICIPANTS (*n* = 23)	

		*n TOTAL*	*VALUE*

Function in IP-LIN (n, %)	Student	15	65.2%

	Professional	8	34.8%

Domain (n, %)	Nursing	19	82.6%

	Social work	4	17.4%

Gender (n, %)	Male	3	13%

	Female	20	87.0%

Mean age (SD)		13	27.2 (12.7)

Highest education level (n, %)	Secondary vocational (1, 2, 3, 4)	5	21.7%

	Higher vocational	2	8.7%

	University	1	4.3%

	Senior general secondary education	6	26.1%

	Lower general secondary education	1	4.3%

In training during the study (n, %)	No	8	34.8%

	Yes	15	65.2%

Current position (n, %)	Teacher higher vocational education	2	8.6%

	Nurse intern	13	56.5%

	Social work intern	2	8.7%

	Social work professional	1	4.3%

	District nurse	4	17.3%

	Practice trainer	2	8.7%

Number of years working in this position, mean (SD)		18	2.9 (6.4)

IP-LIN = Interprofessional Learning and Innovation Network.

Ten interprofessional case discussions were held regarding ten different vulnerable older persons living in the community and dealing with a complex situation. In one case discussion, the older person participated and in another case discussion, the representative of the client’s council of the care organisation participated. On average, almost ten (mean 9.9) participants took part in each of the case discussions (range 6–13). The average duration of a case discussion was 1 hour and 20 minutes (mean 79.8 min). In addition, two focus groups were held, one with four (social work teacher, student nursing, nurse, social worker) and another with five participants (nursing teacher, social worker, student nursing, student social work, nurse), with durations of one hour and 36 minutes, and one hour and 22 minutes, respectively.

### Block 1: Collaborate and understand

During the case discussions, participants exchanged perspectives on potential collaboration between nursing and social care professionals and clarified the roles of various professionals within the neighborhood. This process facilitated mutual acquaintance and enhanced understanding of each other’s responsibilities. The issue of *delineating responsibility* was frequently addressed, exemplified by complex client-centered questions such as: “Until when is a client with dementia permitted to drive?” and “Which professional — the nurse, social worker, or general practitioner — is responsible for discussing this with the client?” No definitive answer was established, as the appropriate approach depended on the client’s specific circumstances and the professionals involved.

It was noted that nurses and social workers *differ in the time they can allocate to clients*. Social workers generally have greater flexibility for client interaction, whereas nurses operate under tighter schedules and time constraints. However, district nurses responsible for specific clients have somewhat more discretion to spend additional time with them. Additionally, social workers often possess deeper knowledge of clients’ personal lives, needs, and preferences. ‘*Communication between the involved professionals’* was another important issue. Interprofessional cooperation hinges on good communication. The client made clear that there are still steps to be taken in this regard. What is common knowledge for nurses is not always so for social work professionals and vice versa. In case discussions, professionals do not automatically ‘*understand each other’s language’*. Nurses and social workers have their *‘own jargon and culture’*. Nurses tend to concentrate on devising solutions and employing evidence-based methods, whereas social workers prioritize problem analysis and catering to client needs, thereby promoting autonomy. Through small-scale quality projects and case discussions, social work and nursing students, teachers and professionals developed interprofessional communication competences. A higher vocational nursing student learned from social workers to listen first to the story behind it.

*“Well yes, I did really build ties with certain clients, and by also listening more to them. Not coming up with solutions but just listening to the story. And getting more insight into a situation. Okay she is dealing with this and I’m just going to offer a listening ear.” (FG; resp. 43)*

And a social work teacher realised:

*“Also very instructive to discover how difficult it is sometimes to seek that cooperation with each other, and to get on the same page with each other. And to listen well and have respect for each other’s area of expertise.” (FG; resp. 24)*

Tension sometimes existed between the two professions, care is bigger and more present than social work. A social work teacher (resp. 25) said:

*“But …. nursing doesn’t have to become social work. And vice versa. So you have to watch out for that too in interprofessional collaboration. And then I stand up for my profession again, of course. Because social work has a less strong professional identity. And also no BIG-registration [Dutch registration for professions in the individual healthcare]. So I’m more afraid of social work being eaten by nursing, to put it bluntly, than the other way around.” (FG)*

Participants discussed working with informal carers (family, neighbors, volunteers) and agreed on their importance in home care. In five cases, professionals initiated *‘cooperation with informal carers’*, but integration into schedules was challenging due to limited consultation. Professionals were cautious about overburdening family and neighbors, sometimes acting as ‘gate-keepers’. The final theme is *‘maintaining client’s autonomy’*. Coordination and consultation should prioritize clients who wish to retain autonomy. In four cases, autonomy was discussed without the client present, focusing on professional responsibility and safety for clients, families, and professionals.

### Block 2: Working out interprofessional care plans

In the case discussions, it repeatedly emerged that people ‘*learned a lot from each other’s perspectives’*: with knowledge about the client, advice and interventions to deal with the sometimes misunderstood behaviours of the client and/or family. At the same time, they discovered that the ‘*lack of a joint care plan for the client to deliver integrated care’* meant that information about the client was not always permanently available to all involved. The case discussions proved to be a good step forward in this respect, but sustainable implementation of the agreed interventions to provide integrated care remained a point of attention. In one case discussion, the client was present, and could be asked directly how he viewed the situation and what was important to him. The ‘*presence of the client was very helpful’* for analysing his problem and was perceived by all as of great added value.

*Practice trainer (resp 5:) “Very nice how we see Mr. taking ownership in this conversation. That he says “I hear a lot of things” and “I want to think about this quietly,” and that’s very instructive for us as professionals and students to remember.” (C6)*

At the same time, ‘*collaborative obstacles’* such as unclear client situations (e.g., domestic violence) and coordination issues (‘Who does what and when?’), impeded clear problem analysis. Despite the lack of a joint care plan, IP-LIN participants experienced better cooperation in delivering integrated care. Nursing and social work professionals and students indicated in the focus groups that they found each other more easily, communication improved, and confidence grew between social workers and nurses, i.e. knowing that the other professional was reliable and competent. They devised interprofessional interventions involving informal care (e.g., joint home visits), broadened their horizons, and developed a more holistic approach. Regarding finding each other, they said:

*Nurse (resp. 18): “Because now, that’s how you know each other too. I now know her [social worker] very well so then it’s easier for me to grab my phone and call her for a consult*.

*Social worker (resp. 19): Yes, and now I can actually always put someone you can take in care. That’s a kind of easy.” (FG)*

### Block 3: Deal with problems

Critical reflection on one’s own actions in the interprofessional case discussions focused on *‘reflection on one’s own attitude towards the client and/or family’*. The case discussions ended with a reflection on the impact of the interprofessional case discussions on the professionals, teachers, and students. One higher vocational nursing student stated:

*Student (resp. 15): “Yes, I also found it very instructive that now you start thinking more about what kind of solution we are going to come up with for this. But also that you look at things differently now such as domestic violence, I was completely unaware of that. So things like that I’ll think more about that next time.”*

*Social worker (resp. 19): “When you are helping with the morning care?”*

*Student (resp. 15): “Yes, so that I can recognise any signs of violence.” (C1)*

They also ‘*reflected on team work’* during the interprofessional case discussions. This way of sharing each other’s work stimulated a growing confidence in each other. A secondary vocational nursing student described:

*“Well just, I now involve the social worker more in certain situations. Because I now know that you’re there, but even when I doubt I now think, well maybe that’s a thingy for you.” (C8; resp. 40)*

Participants reflected on interprofessional team work during the case discussions, which led to the conclusion that these talks had a positive impact on delivering integrated care to client and/or family. A higher vocational nursing student said:

*“I know quite a lot about this client. But I like the fact that we can now really do something for him. Instead of just talking about it a bit, I obviously do talk about this situation with colleagues and supervisor. But now at least we really have something we can do. As a follow-up step to help him, yes I like that.” (C3; resp. 3)*

Case discussions revealed moral dilemmas such as providing care when clients resist and balancing autonomy with safety. The interprofessional conversations offered valuable insights and support, but implementation of proposed solutions was inconsistent due to changing client situations or rejection by nursing teams. Continuity was also difficult to maintain, staff turnover in nursing, social work, and among interns, hindering sustainable implementation. Challenges in addressing these issues therefore remain.

### Block 4: Make appropriate referrals

Block 4 was mentioned less often, but the local network of social work and health services was frequently discussed. In addition to care and social work professionals, clients can access various community supports — such as home support, day care, case management, domestic help, occupational therapy, and meal delivery. These services are ‘*important partners in providing integrated care’* for independently living clients and are therefore valuable to include in case discussions. A social worker expressed:

*“Yes, for me it would be very helpful if other support partners also participated in the case discussions. Then I find it very nice that I know who I can reach to just spout off and asking help. What to do, what not to do. I find it nice that we have short lines of communication.” (FG; resp. 13)*

Participants indicated that involving other professionals was desirable, but they were unable to put this into practice. While involvement does not always require formal referrals, ideally the client’s needs should determine which professionals join case discussions.

### Block 5: Evaluate

As described before, ‘*not all interventions were implemented’* by nurses and social workers, and care plans were ‘*not always methodically evaluated with the client’*. Teams often relied on informal discussions with clients and families. Systematic evaluation was hindered by factors like client admission to nursing homes or psychiatric issues. However, when clients participated in case discussions, evaluations were very positive, enhancing client well-being (e.g., moving bed and TV, going with a volunteer for small errands). From the 7th case discussion, the team aimed to evaluate previous agreements, but this was not always done methodically or with all involved parties. A practice trainer phrased this as:

*“I wonder if we followed up enough. I did have the idea that the people who participated in the discussions were alert to follow through on the things that had been agreed. However, I wonder if it was passed on correctly to others in the team, and whether it got into the care plans properly. But that we did better and better. However, we often saw that the professionals no longer knew very well what had been agreed, and whether something had been followed up. So professionally, there is still something to be improved.” (FG; resp. 5)*.

## Discussion

This study offers insights into how nursing and social care professionals, students, and teachers collaborated and learned within an interprofessional Learning and Innovation Network (IP-LIN). The interprofessional mix in case discussions shifted the focus toward clients’ abilities rather than their health problems, largely due to the added perspective of social workers. Participants recognised and developed their interprofessional competences, gaining a better understanding of integrated care, though implementation often lagged. Key barriers included limited involvement of all relevant support partners, insufficient follow-up on agreements, the time required for interprofessional collaboration, and the difficulty of working methodically without a shared care plan.

Involving clients and informal carers in case discussions was a positive experience for all participants, including the clients themselves. Although facilitating their (online) participation was sometimes challenging, it proved achievable with support. They play an important role in the interprofessional team, even as non-professionals. Contrary to nurses’ and students’ expectations, the client’s main request focused on maintaining independence — a social-wellbeing need that offered valuable learning for everyone involved. Independence is one of the most important issues for older adults. They feel it is their own responsibility to resolve issues in the psychological and social domain of health and they prefer not to ask for help for these types of problems [29,30]. So having an interprofessional case discussion in which both care and social work participate and in which more time is taken, may provoke clients to be more open about their needs. The positive experience of the client participating in the case discussion was a great lesson the IP-LIN participants learned on ‘social prescribing’ [31], which is a low cost intervention and empowers clients as people based on what matters to them. Social prescribing links health and social care and ensures shared responsibility, which reduces the pressure on clinical care by deploying appropriate support and prevention [31].

IP-LIN professionals often objected to involving clients or informal carers in case discussions, arguing that clients were too vulnerable or families overburdened. A Finnish study shows that nurses commonly classify clients as ‘knowledgeable clients’, ‘health shoppers’, or ‘vulnerable clients’. The latter group includes people with long-term illnesses, older adults with comorbidities or memory problems, older carers in poor health, people with mental health issues, and those with limited language skills. Subsequently, the nurses positioned themselves as advocates acting on behalf of these clients [32]. Nurses sometimes decide for rather than with the client, yet even vulnerable clients value having choices. Supporting patients with complex needs requires integrated care and better access to information about available support options that match their needs [33]. Participating in case discussions provides an opportunity to discuss various choices and give clients more autonomy. Also, increasing patient choice can be expected to have an effect on the patient-provider relationship and on the content of work and responsibilities of nursing and social workers [33]. Professionals may need to be better facilitated and invest more time to explore with clients and informal carers how to participate in such (online) case discussions.

Applying the cycle of methodical work, as well as discontinuity of participating professionals and students in case discussions and the lack of a joint care plan in particular proved most troublesome to deliver integrated care. In the Netherlands and other European countries, care and social work organisations also use different digital client record systems, further complicating integrated care. This situation hinders integrated care [34], and can be seen as a wicked problem: a problem in which many different stakeholders are involved and many different factors are at play, e.g. privacy rights, human behaviour, technological, and cultural factors. Interprofessional learning networks are well suited to complex issues without easy answers, where multiple parties — including the client or their representative — are needed to reach a solution [35]. Learning networks, like LINs, CIUs, but also DEUs and NDUs create space for working on such problems i.e. by reflection and learning between all involved professionals, students and teachers about the research, the joint practices, the necessary preconditions, intended outcomes and implementation, which enables improved quality of care [15,16,18,19]. Here, it is important to take time for (interprofessional) reflection so that the client’s questions are understood, and all involved learn to work together across professional boundaries. This helps them understand each other’s language, build mutual interest, align on shared goals, and deliver integrated care [21,35]. Despite the advantages of learning networks, there are also some issues. From the IP-LIN could not be demonstrated that the quality of care and support for older adults living in the community improved. This question, ‘how can we claim that clients benefit from learning networks?’ remains open [18]. However, that is the larger goal of setting up a learning network. Another issue is that the structure of learning networks is often missing. The quality projects where various stakeholders collaborate for example, students investigate the same problems year after year. Repeatedly the stakeholders ultimately turn out not to have the capacity, resources and opportunities to solve the problems they encounter. Substantial time and resources must be made available by administrators, civil servants and politicians so that professionals, clients and other partners in the learning network can actually work on structural solutions to problems and practice change [17,18,19,35,36]. While the bottlenecks of learning networks should not be ignored, they can be of great significance when it comes to exchanging between education and practice awareness, increased self-confidence, improved interprofessional collaboration, development of new practices, implementation of innovations, and on team performance in terms of effectiveness, efficiency and innovativeness [15,16,18,37].

### Limitations

This study has strengths and limitations. One limitation is that this case study was performed with qualitative data. These data are very informative about the participants’ behaviour and thoughts, and gave us great insights into the interprofessional collaboration. However, they cannot be generalised due to the sampling and research strategy of the study, the specific setting, and the relatively small number of participants in the case discussions and focus groups. A strength is that the used analysis model, the interprofessional competences model [25], closely matched with the research questions, but afterwards it may have been informative to also use the CFIR-model [25] for analysing the data. This model would have potentially added value in gaining more insight into the implementation of the interprofessional collaboration within the IP-LIN. The final limitation is that owing to the pandemic, seven of the ten case discussions and focus groups were held online. Although, this was not the subject of the study, online focus groups have advantages and disadvantages [38]. Participation is easier for hard-to-reach target groups, such as adults with physical disabilities. Busy nurses were also able to participate more easily. The main disadvantage of online case discussions are the lack of personal contact between respondents among themselves and in interaction with the moderator.

## Lessons learned

- The interprofessional collaboration within the IP-LIN gave the professionals, students, and teachers the opportunity to get to know each other better and learn about each other’s tasks, responsibilities and perspectives. Working and learning together leads to greater mutual trust, which led to improved collaboration.
- It proved complicated to apply the cycle of methodical work in interprofessional collaboration. Important hurdles were: discontinuity of participating professionals and students in case discussions, and the lack of a joint care plan to deliver integrated care, which is an international issue.
- Because far more nurses than social workers are involved in caring for vulnerable older adults, the nursing discipline was more strongly represented in the IP-LIN. Yet both professions are expected to value each other’s expertise. Integrating social and physical aspects of care requires recognising both roles and providing care jointly. Effective interprofessional work depends on equivalency and full participation of all stakeholders. An IP-LIN therefore needs connection to diverse professional perspectives, extending beyond nursing and social care. This calls for a broader network, transparency, and awareness of differing goals, interests, competencies, and available services.
- Case discussions within an IP-LIN are instructive for all participants, including the client and their informal carer. Despite the fact that older clients living in the community are often vulnerable, they would like to have a say regarding their care and support plan. Professionals should be aware of this need for clients and informal carers participation but also of their own tendency to act as advocates for the clients. Given the big task, the double ageing [1] we face in the Netherlands, we need to look differently at client and informal carer participation. This calls for a renewed commitment to fostering genuine dialogue and shared decision-making that respects the voices of older clients and their informal carers, ultimately enhancing the quality and responsiveness of integrated care.
- IP-LINs offer several benefits and drawbacks for various stakeholders, including professionals, students, educators, and clients or citizens. On the positive side, the activities within IP-LINs promote the growth of interprofessional skills. It is crucial for students and professionals to cultivate an ‘interprofessional’ mindset, which encourages them to engage in dialogue with other professionals, seek collaborative opportunities, and value informal care and support. This approach fosters cooperation with the understanding that no one, whether a professional or a client, should be isolated. However, a potential challenge is ensuring that each new learning network does not become a one-time initiative. To maintain the long-term implementation of the outcomes achieved, it is essential to involve administrators, civil servants, and politicians in the ongoing development of these learning networks from the outset.

## Conclusion

This case study aimed to gain insight into interprofessional learning and collaboration between care and social work professionals, students and teachers within the IP-LIN, to discover influencing factors and to find out to what extent this IP-LIN contributes to integrated elderly community care. Influencing factors are:

- Working and learning together leads to greater mutual trust, which led to improved interprofessional collaboration.
- Turnover of professionals and students in case discussions and the lack of a joint care plan hinders integrated care and a methodical way of working.
- For successful interprofessional learning and working, participation of all professionals involved with the client (beyond the scope of nursing and social care) is necessary. This requires an extended network, transparency, and awareness regarding differing goals, interests and competences needed, as well as knowing what services are available.
- Clients and informal carers must be actively involved in integrated case discussions, as they wish to participate in decisions concerning their care and support.
- Interprofessional learning networks are valuable for students, teachers, professionals, their administrators and civil servants who want to invest in the development of interprofessional competences among nursing and social care professionals, leading to better quality of care. However it is important to ensure that the results achieved within the learning network are implemented on a structural basis. Therefore, involve administrators, civil servants and politicians in the development of learning networks from the outset and on an ongoing basis, and train students at school in an interprofessional attitude.

Students, teachers and professionals from social work and nursing broadened their perspectives and developed their interprofessional competences during the interprofessional collaboration within the IP-LIN, but there is still a lot to learn. Interprofessional learning is a prerequisite for delivering integrated care. Professionals and students believed that the client living in the community benefited from interprofessional collaboration and well-integrated care. However, it cannot be concluded from this study that interprofessional collaboration improved the client’s quality of life. Integration of care and support will be of great value, but there are still hurdles to overcome in daily practice.

## Additional File

The additional file for this article can be found as follows:

10.5334/ijic.9122.s1 Supplemental Material. Topics of the focus groups, derived from CFIR-model.

## Data Availability

The data underlying this article are available in a public, open access repository, and can be accessed at *DANS* https://doi.org/10.17026/dans-xma-xv8e.

## Note

Integrated care in this article refers to integrated nursing and social care.
