# Supplementary material for: Lessons Learned from Providing Integrated Care Within an Interprofessional Learning and Innovation Network in the Community
Source: Int J Integr Care. 2026 Jun 25;26(2):22. doi: 10.5334/ijic.9122 (PMC13308522; doi:10.5334/ijic.9122)
Supplement: Supplemental Material. — Topics of the focus groups, derived from CFIR-model. [file ijic-26-2-9122-s1.pdf]

Supplementary material

Fourteen discussed statements in the focus groups

- Interprofessional collaboration gives me insights from a different angle.
- Interprofessional cooperation encourages me to think critically about my work and my attitude.
- Agreements we make with each other as professionals in case discussions are followed up and evaluated.
- An IP-LIN gives me additional workload.
- Interprofessional working increases my job satisfaction.
- There are no obstacles to interprofessional work from now on.
- I came across many promoting factors that made this IP-LIN work well.
- I want to be part of an interprofessional team.
- Because of our integrated approach in the IP-LIN (care and social work), people stay at home for longer than before.
- When I have a dilemma which is outside my area of expertise, I have been able to seek contact with another discipline more easily since I have been part of this IP-LIN.
- To provide integrated care, an integral care and support plan is necessary.
- This interprofessional team has given me new insights into the care and support of vulnerable older people living in the community.
- In our IP-LIN, clients have received better care and support over the past 2 years than they would have received before.
- Social work and nursing should belong to the same organisation in the district.

Interprofessional competence model and interprofessional building blocks

Building blocks	Items
1. Collaborate and understand	1.1 Initiate mutual working relationships and take responsibility to act supportively and assist IP team members<br>1.2 Sustain working relationships in IP teams<br>1.3 Describe distinct roles and responsibilities in IP teams<br>1.4 Describe individual and professional expertise<br>1.5 Describe responsibilities and competences of other professionals and acknowledge professional boundaries<br>1.6 Show insights, understanding and respect for the roles, responsibilities and competences of other professionals in relation to the own expertise/profession<br>1.7 Facilitate other professionals in the IP team to provide care so that individual clients benefit by maximal health gains<br>1.8 Understand how to cope with differences, misunderstandings, opposite positions and shortcomings of IP team members<br>1.9 Know when, how and why other healthcare workers with different backgrounds need to be involved in IP care<br>1.10 Apply information of other professionals in profession-specific interventions<br>1.11 Fulfil a role in IP teams as perceived satisfactory by IP team members
2. Work out	2.1 Make client information and/or other relevant documents

interprofessional care plans	accessible to other professionals to optimize care<br>2.2 Provide complete client information from a professional-specific perspective<br>2.3 Participate in a shared problem analysis with relevant professionals involved whereby the clients' individual perspective is central<br>2.4 Formulate, together with clients/family members and other professionals, care and treatment goals<br>2.5 Make decisions on client-centred care and treatment goals together with clients/ family members and other professionals<br>2.6 Deliberate together over and make decisions with clients/family members and other professionals about the client-centred care and treatment plan and confirm it<br>2.7 Make sure that clients/family members understand and agree on the IP care plan<br>2.8 Collaborate with clients/family members and other professionals to implement an IP care plan
3. Deal with problems	3.1 Reflect on one's own behaviour in the context of the IP team<br>3.2 Provide constructive and appropriate feed-back to IP team members<br>3.3 Reflect on IP teamwork<br>3.4 Identify individually perceived problems and dilemmas and discuss them in the IP team<br>3.5 Identify and articulate problems within IP teams<br>3.6 Participate in the solving of IP team problems<br>3.7 Anticipate individually perceived problems in IP teams<br>3.8 Discuss and solve individually perceived problems in the context of IP teamwork
4. Make appropriate referrals	4.1 Describe the network of social and health services in certain geographical areas and interact with them<br>4.2 Make appropriate referrals to different professionals within the IP team and within relevant other professionals, external to the IP team<br>4.3 Use a language level and style that is understandable to clients/family members and other professionals when referring (written, verbal, digital, by phone etc.)<br>4.4 Provide relevant and necessary information during handovers and referrals<br>4.5 Apply accessible tools for the documentation in client records and referrals
5. Evaluate	5.1 Evaluate client-centred care with clients/family members in the role of individual healthcare worker, in the context of an IP team and within integrated care pathways<br>5.2 Evaluate the communication with clients/family members, team members, professionals in integrated care pathways and advocacy groups (social and healthcare)<br>5.3 Evaluate the IP decision making with clients/family members, team members, professionals in integrated care pathways and advocacy groups (social and healthcare)<br>5.4 Evaluate the efficiency of IP care with clients/family members, team members, professionals in integrated care pathways and advocacy groups (social and healthcare)<br>5.5 Adjust the care provided by an IP team based on the

	evaluation with clients/family members, team members, professionals in integrated care pathways and advocacy groups (social and healthcare)<br>5.6 Contribute actively and professionally to IP case meetings, team appointments, team collaboration and networking
